# A Rare Clinical Presentation of Cholangiocarcinoma

**DOI:** 10.1155/2017/7156838

**Published:** 2017-12-13

**Authors:** Elisa Gravito-Soares, Marta Gravito-Soares, Pedro Figueiredo, Luis Tomé

**Affiliations:** ^1^Gastroenterology Department, Centro Hospitalar e Universitário de Coimbra, Coimbra, Portugal; ^2^Faculty of Medicine, University of Coimbra, Coimbra, Portugal

## Abstract

Cholangiocarcinoma is an uncommon tumor, often diagnosed in the context of obstructive jaundice. Brain metastasis rarely occurs with the cerebellum being a rare site of spread of this type of tumor. Few cases of cholangiocarcinoma have been reported in the literature and this type of tumor is associated with a very poor outcome. We present a very rare form of clinical presentation of cholangiocarcinoma with neurologic symptoms due to cerebellar metastases.

## 1. Introduction

Cholangiocarcinoma is a rare tumor of the bile duct, often diagnosed in the context of obstructive jaundice [1–3]. Usually, patients with cholangiocarcinoma have a locally advanced disease at the diagnosis with distant metastasis being uncommon [1]. Brain metastasis is rare and associated with poor prognosis [2].

Very few cases of brain metastasis due to primary cholangiocarcinoma [3–8] have been reported in the literature and there is only one case with neurologic symptoms at presentation [3]. In this work, we describe the case of a patient with headache, as initial symptom of an extrahepatic cholangiocarcinoma with cerebellar metastasis.

## 2. Case Report

**Figure 1:**
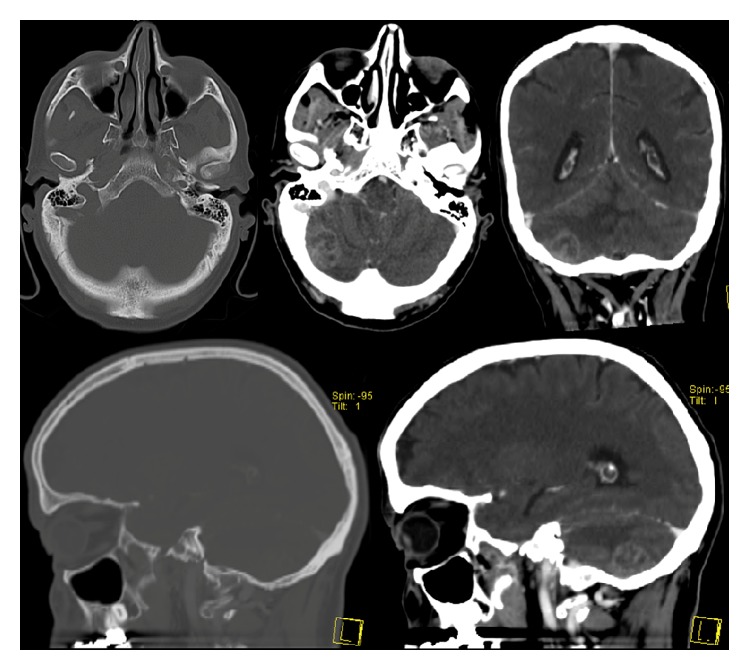
Brain computed tomography scan (bone and brain windows) showing one lesion in the right cerebellum with heterogeneous contrast enhancement.

**Figure 2:**
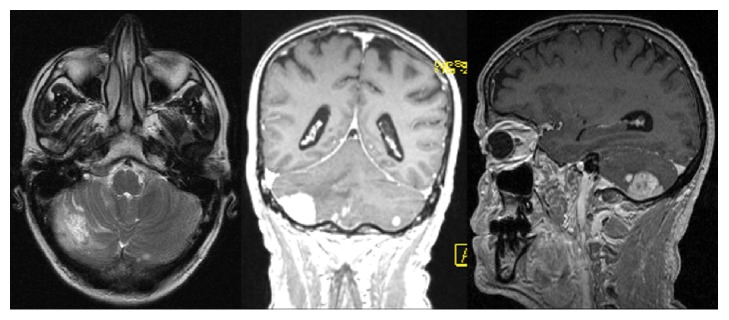
Magnetic resonance imaging showing multiple bilateral lesions in the cerebellum with strong and irregular contrast enhancement, central necrosis, and mass effect.

**Figure 3:**
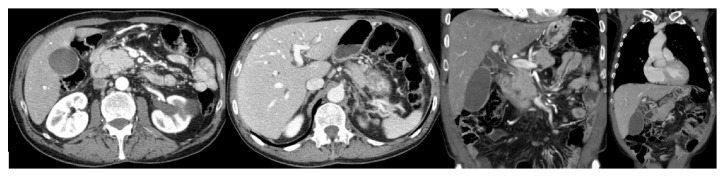
Cervical-thoracic-abdominal-pelvic computed tomography revealed parietal contrast enhancement without intraluminal well-defined mass at the distal common bile duct level with upstream dilatation of the extrahepatic and intrahepatic bile ducts.

**Figure 4:**
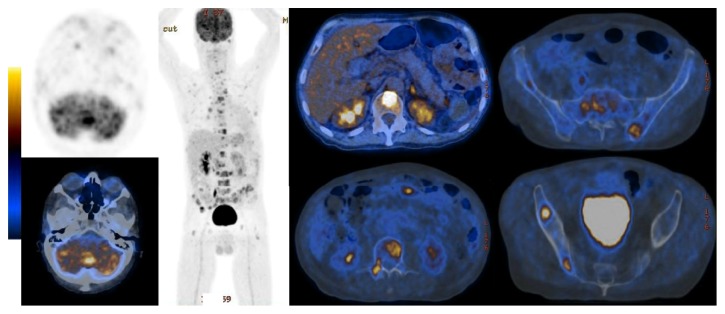
18-Fluorodeoxyglucose positron emission tomography revealing a hypermetabolic lesion at the common bile duct/duodenum topography with cerebellar, thoracic-abdominal lymph nodes, left suprarenal gland, and bone metastasis.

An autonomous 66-year-old Caucasian man with past medical history of arterial hypertension and social alcohol consumption (15 g/day) was admitted with two weeks of nausea, vomiting, and jaundice. About 4 months earlier, the patient started daily occipital headache with frequent recurrence to the emergency department, misdiagnosed as uncontrolled arterial hypertension. There was no history of blood transfusions, recent trips, over-the-counter drugs, prior infectious diseases, or jaundice episodes. Physical examination was unremarkable except for mucocutaneous jaundice and mild discomfort in the upper right quadrant of the abdomen. Neurologic examination revealed no significant abnormalities, including cerebellar signs such as vertigo, ataxia, nystagmus, dysmetria, dysarthria, dysdiadochokinesia, or other signs. Laboratory analysis showed lactate dehydrogenase 268 U/L (125–220 U/L), aspartate aminotransferase 150 U/L (<31 U/L), alanine aminotransferase 316 U/L (<34 U/L), alkaline phosphatase 955 U/L (40–150 U/L), gamma-glutamyl transferase 1465 U/L (<38 U/L), total bilirubin 4.5 mg/dL (0.3–1.2 mg/dL), and direct bilirubin 3.2 mg/dL (0.1–0.5 mg/dL). Serum albumin, serum amylase, and prothrombin time were normal. Tumor marker CA 19-9 was elevated (299 U/mL; <37 U/mL) and alpha-fetoprotein and carcinoembryonic antigen (CEA) were normal. An extensive etiological workup was performed excluding liver diseases, infections, autoimmune conditions, and risk factors for cholangiocarcinoma. Abdominal ultrasound showed dilatation of the common bile duct (12 mm) and intrahepatic ducts and gallstones. Esophagogastroduodenoscopy was normal. Computed tomography scan (CT) of the brain revealed one mound-shaped lesion with biconvex dural contact in the right cerebellum with heterogeneous contrast enhancement (Figure 1). Other causes than neoplastic lesion were ruled out, including cryptococcosis, toxoplasmosis, syphilis, or human immunodeficiency virus infection. Subsequently, for a better characterization of the cerebellar lesion, brain magnetic resonance imaging (MRI) was performed revealing multiple bilateral lesions with strong and irregular contrast enhancement, central necrosis, and mass effect, suggesting cerebellar metastases (Figure 2). In order to find out the primary site of this tumor, a thorough examination of the cutaneous tegument was performed, excluding melanoma. Colonoscopy was normal. Cervical-thoracic-abdominal-pelvic CT confirmed dilatation of intrahepatic and extrahepatic bile ducts with progressive narrowing of the common bile duct at the prepancreatic level. The narrowed segment of the bile duct showed a parietal contrast enhancement without well-defined intraluminal mass (Figure 3). Thus, endoscopic retrograde cholangiopancreatography (ERCP) was attempted. However, this procedure revealed being impossible to perform due to edema and congestion of the duodenal mucosa with pearly-white appearance, resulting in luminal stricture. Biopsies showed a lymphovascular permeation of the duodenum due to a poorly differentiated adenocarcinoma with positive immunohistochemical staining for CK7 and CK19, consistent with a primary biliary neoplasm. Positron emission tomography scan (PET) with 18-fluorodeoxyglucose was performed revealing a hypermetabolic lesion at the common bile duct/duodenum topography with cerebellar metastasis and, additionally, other secondary lesions in the thoracic-abdominal lymph nodes, left suprarenal gland, and multiple bone sites (Figure 4).

The patient started systemic steroids with initial improvement of neurologic symptoms. However, subsequently neurological worsening was verified with gait ataxia and vertigo and therefore brain radiotherapy was started after a multidisciplinary therapeutic decision meeting. The patient died within six weeks of diagnosis, having performed only one session of whole-brain radiotherapy (WBRT).

## 3. Discussion

Cholangiocarcinoma is a rare tumor and the most common malignancy of the biliary tract. Its incidence has been continuously increasing and usually develops in the setting of progressive cholestasis and chronic inflammation [1, 2]. However, the patient did not present cholangiocarcinoma risk factors, including parasitic infections, primary sclerosing cholangitis, biliary-ducts cysts, hepatolithiasis, toxins, or other less-established risk factors such as inflammatory bowel disease, hepatitis C or B, liver cirrhosis, diabetes, obesity, significant alcoholism (>40 g/day), or smoking [1, 9]. Despite a mean age of 50 years for the onset of symptoms, diagnosis is usually made at the age of 65. Based on the anatomic site of origin, cholangiocarcinoma is classified as intrahepatic, perihilar, and extrahepatic with distinct tumor biology, clinical presentation, and management. Extrahepatic cholangiocarcinoma is the second most common subtype (27–42%) with a mild male predominance. It can arise as mass-forming type, periductal infiltrating type, or intraductal growth type, usually with obstructive jaundice or associated complications, such as cholangitis and mass effect symptoms [1, 3]. CT and MRI present a high diagnostic accuracy (up to 93%). PET can be useful in cases of metastatic disease of unknown primary tumor [1]. ERCP provides additional diagnostic value in the assessment of strictures, cytological analysis, and stent-placement for biliary decompression [1]. Elevated CA 19-9 favors the diagnosis of cholangiocarcinoma, after other malignant, inflammatory, and infectious diseases of hepatobiliary-pancreatic system have been excluded [1]. A recent study that assessed the diagnostic and prognostic value of CA 19-9 and CEA in cholangiocarcinoma verified that more than 50% of patients with cholangiocarcinoma had normal CEA level and high CA 19-9 level (median of 103.0 U/L). This cut-off for CA 19-9 had prognostic value, being associated with a higher probability of metastasis and lower curative treatment rates [9].

In early stage of cholangiocarcinoma, Whipple surgery represents the main treatment. However, most patients present with advanced disease and therefore curative treatment is almost never feasible. Given the intrinsic resistance to radiation and chemotherapy, the prognosis remains dismal with a 5-year survival rate of 27–37% [1]. Thus, most patients only benefit from symptom-based supportive care.

Cholangiocarcinoma often spreads to the regional lymph nodes and adjacent organs. Distant metastasis is rare with bones, muscles, and thyroid gland being the most frequently involved sites. Brain metastasis is extremely rare with very few cases [3–7] and one case series reported in the literature that showed 0.15% of incidence [2]. Since brain imaging is not performed routinely, but only in the presence of symptoms related to space-occupying lesions (headache, motor weakness, gait disturbance, or altered mental status), the real prevalence of brain metastasis is not well established [2]. Furthermore, this tumor is poorly vascularized making hematogenous mechanism of spread unlikely. Regarding brain metastasis, the exclusive involvement of posterior fossa is also rare, reported only once [3, 7]. Usually, the distribution of brain metastases follows the relative weight of each area (80% in the cerebral hemispheres, 15% in the cerebellum, and 5% in the brainstem) [8]. The mechanism of cerebellar metastasis in cholangiocarcinoma is not yet known [3]. Published literature has only described that gastrointestinal tumors have an unknown predilection to metastasize to the posterior fossa, maybe related to spread via Batson's venous plexus [8].

Chindaprasirt et al. described the only existent case series of brain metastasis by cholangiocarcinoma. They showed that all eight cases were intrahepatic or perihilar tumors with the median age of 60 years. Diagnosis was based on neurologic symptoms in all patients, three of whom received WBRT and one underwent surgery. The median survival after diagnosis of brain metastasis was 9.5 weeks [2], being worse than other metastatic brain tumors [10, 11].

As far as we know, brain metastasis has never been reported in extrahepatic cholangiocarcinoma. In addition, this is the second reported case of initial neurologic symptoms and brain metastasis exclusively to the posterior fossa [3].

In conclusion, we report the case of an atypical presentation of extrahepatic cholangiocarcinoma with neurologic symptoms related to cerebellar metastasis, a rare site of spread. The prognosis was very poor, similar to other locations of cholangiocarcinoma. Therefore, clinicians should consider the possibility of brain spread in any type of cholangiocarcinoma, including extrahepatic location. Neurologic symptoms may precede hepatobiliary dysfunction.

## Abbreviations

CEA: Carcinoembryonic antigen
CT: Computed tomography scan
MRI: Magnetic resonance imaging
ERCP: Endoscopic retrograde cholangiopancreatography
PET: Positron emission tomography scan
WBRT: Whole-brain radiotherapy.
